# Genomic Sequence around Butterfly Wing Development Genes: Annotation and Comparative Analysis

**DOI:** 10.1371/journal.pone.0023778

**Published:** 2011-08-31

**Authors:** Inês C. Conceição, Anthony D. Long, Jonathan D. Gruber, Patrícia Beldade

**Affiliations:** 1 Instituto Gulbenkian de Ciência, Oeiras, Portugal; 2 University of California Irvine, Irvine, California, United States of America; 3 Institute of Biology, Leiden University, Leiden, The Netherlands; Ecole Normale Supérieure de Lyon, France

## Abstract

**Background:**

Analysis of genomic sequence allows characterization of genome content and organization, and access beyond gene-coding regions for identification of functional elements. BAC libraries, where relatively large genomic regions are made readily available, are especially useful for species without a fully sequenced genome and can increase genomic coverage of phylogenetic and biological diversity. For example, no butterfly genome is yet available despite the unique genetic and biological properties of this group, such as diversified wing color patterns. The evolution and development of these patterns is being studied in a few target species, including *Bicyclus anynana*, where a whole-genome BAC library allows targeted access to large genomic regions.

**Methodology/Principal Findings:**

We characterize ∼1.3 Mb of genomic sequence around 11 selected genes expressed in *B. anynana* developing wings. Extensive manual curation of *in silico* predictions, also making use of a large dataset of expressed genes for this species, identified repetitive elements and protein coding sequence, and highlighted an expansion of *Alcohol dehydrogenase* genes. Comparative analysis with orthologous regions of the lepidopteran reference genome allowed assessment of conservation of fine-scale synteny (with detection of new inversions and translocations) and of DNA sequence (with detection of high levels of conservation of non-coding regions around some, but not all, developmental genes).

**Conclusions:**

The general properties and organization of the available *B. anynana* genomic sequence are similar to the lepidopteran reference, despite the more than 140 MY divergence. Our results lay the groundwork for further studies of new interesting findings in relation to both coding and non-coding sequence: 1) the *Alcohol dehydrogenase* expansion with higher similarity between the five tandemly-repeated *B. anynana* paralogs than with the corresponding *B. mori* orthologs, and 2) the high conservation of non-coding sequence around the genes *wingless* and *Ecdysone receptor*, both involved in multiple developmental processes including wing pattern formation.

## Introduction

Accumulation of genomic sequence data for different species is allowing an in-depth understanding of genome properties and evolution. Analysis of whole genomes of target species enables a detailed characterization of genome content and structure, and comparative analysis of genomic sequence across species provides insights about different aspects of genome dynamics and evolution (e.g., [1]). Widening phylogenetic representation of genomic data has allowed *in silico* identification of new protein-coding and miRNA genes, and regulatory sequence (e.g., [2], [3]). However, and despite the increasing number of eukaryotic assembled genomes in the public depository [4], we are still far from representative coverage of biological diversity. This is especially so for groups with larger genomes whose full sequencing still requires a significant investment, and/or where repetitive or polymorphic sequence renders genome assembly a bioinformatic challenge.

Bacterial Artificial Chromosomes (BACs), where large (typically around 150 Kb) fragments of genomic DNA are cloned and can be accessed individually, are a valuable resource for species where a complete genome sequence is not (yet) available. They allow focus on particular genomic regions (often around genes of interest), and have been used successfully for different ends such as sequence annotation (including access to gene coding and regulatory regions, physical mapping, development of genetic markers, analysis of synteny, or to assist whole genome assembly (e.g., [5], [6]). BAC libraries are available for many species, including different lepidopterans (the insect order of butterflies and moths) which have relatively large and typically repetitive genomes [7]. This is one of the most diverse groups of animals and includes many agricultural pests and one of only two domesticated insects, the silkworm *Bombyx mori*.

The Lepidoptera have an unusual set of genetic properties, combining holocentric chromosomes, heterogametic females, and male-restricted meiotic recombination, whose consequences for genome evolution remain largely unexplored. The genome of *B. mori* is completed [8], [9] and provides an invaluable reference for comparative genomics in this group (e.g., [10], [11]). However, this is the only genome publicly available for this relatively vast and ancient group, with more than 150,000 described species [12], [13]. Butterflies have diverged from moths some 140 MYA [14] and, despite growth in genomic resources [15], no full genome sequence has yet been made available for any species in this group. Butterflies have interesting biological properties (such as color vision and novel wing color patterns) and include many textbook examples of studies in ecology and evolution – for example, long distance migrations of monarchs [16], mimicry in *Papilio* and *Heliconius* [17], [18], [19], mutualistic relationships between lycaenids and ants [20], and wing pattern plasticity and evo-devo in *Bicyclus* and *Junonia* [21]. *Bicyclus anynana* has been established as a butterfly model in the study of the evolution and development of wing color pattern elements called eyespots [21], [22], [23], [24]. This species has a large collection of expressed gene sequences and the densest gene-based linkage map available to date for any butterfly species [25], [26]. A BAC library available for *B. anynana* [27] allows access beyond the coding regions of genes of interest, including genes involved in wing pattern formation.

Here, we analyze large genomic regions in BAC clones selected for containing 11 genes expressed during *B. anynana* wing development, at stages relevant for color pattern formation [25]. The selected genes include those encoding signaling molecules proposed as candidate morphogens in the induction of eyespots (Decapentaplegic, Dpp, and Wingless, Wg; [23]) as well as some of their regulators possibly responsible for pattern variation (APC-like, APC, and Naked cuticle, Nkd; [28]), transcription factors implicated in eyespot ring patterning (Distal-less, Dll, and Engrailed, En; [29], [30], [31]) as well as other transcription regulators (Apterous, Ap, and DP transcription factor, Dp), enzyme Vermilion (V) presumably involved in pigment synthesis on developing wings [32], Ecdysone receptor (EcR) involved in wing pattern plasticity [33], [34], and the antioxidation gene *Superoxide dismutase 2* (*Sod2*; [35]). Our annotation of the BAC sequences enabled the identification of repetitive DNA and transposable genetic elements, and prediction of putative protein-coding genes, including the 11 target genes as well as the genes around them. The comparative analysis to orthologous regions in other lepidopteran species allowed us to assess fine-scale conservation of gene order (synteny) and also of nucleotide sequence in predicted protein coding and non-coding DNA.

## Results and Discussion

**Figure 1:**
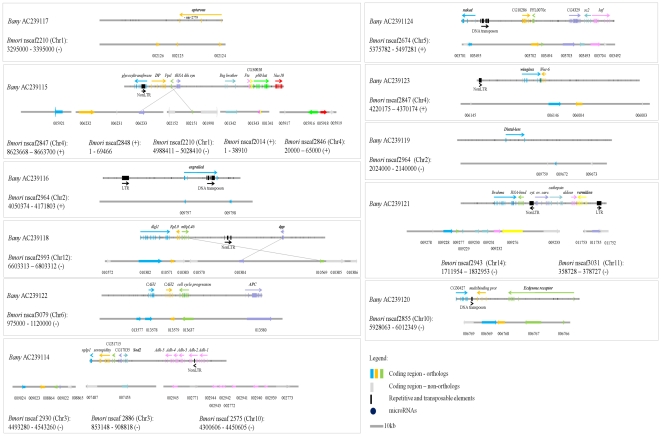
Annotation of *B. anynana* genomic regions and fine-scale synteny with *B. mori*. Each *B. anynana* BAC sequence is represented, with the corresponding scaffold in *B. mori* (including information on chromosomal location). Each putative gene is represented by a different color: *B. anynana* gene names in bold correspond to those on which BAC selection was based (Table 1), and *B. mori* gene names reflect SilkDB annotation (e.g., **010572** is SilkDB gene BGIBMGA**010572**). Exons are explicitly annotated for *B. anynana* as stripes of the same color (darker shade for duplicated exons). Arrows indicate the direction of transcription of each gene, and fine lines are used for highlighting chromosomal rearrangements. The figure contains a legend for the representation of sequence length, and for the protein-coding genes, repetitive sequence, transposable elements, and microRNA identified in this study. Details on all *B. anynana* predicted peptides can be found in Table S3.

**Figure 2:**
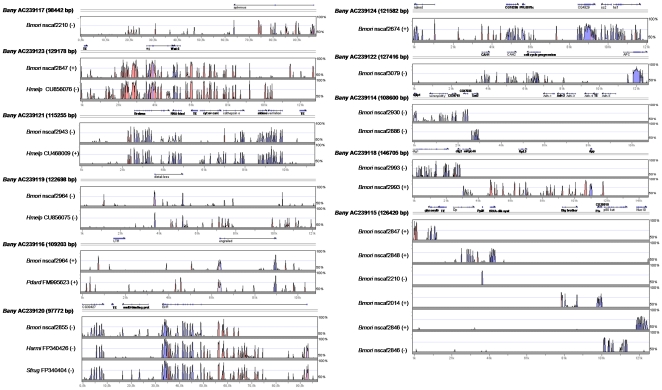
Conservation of DNA sequence in relation to other lepidopterans. VISTA plots of all BAC sequences against *B. mori* and, when available, other lepidopterans (moths *Bombyx mori*, *Helicoverpa armigera*, *Spodoptera frugipera*, and butterflies *Papilio dardanus*, *Heliconius melpomene*). Regions more than 70% conserved in a 100 bp window (VISTA default settings) appear as peaks with blue corresponding to annotated protein-coding regions and red to conserved non-coding sequence. Figure S2 shows close-up and extended analysis of regions around genes *wingless* and *Ecdysone receptor*.

We analyzed ∼1.3 Mb of genomic sequence for the butterfly *Bicyclus anynana*, an emerging model in the study of wing pattern evolution and development [36]. This sequence was part of 11 BAC clones selected (from a library available for the species [27]) for containing 11 genes (Table 1) expressed in developing wings during the stages relevant for color pattern formation [25]. Assembled BAC sequences were characterized and annotated in relation to a number of criteria (see Methods), including detection of repetitive elements and prediction of protein-coding genes (Table 1, Figure 1). Comparison of gene content with the available gene-based linkage map of *B. anynana* [26], shows that the BACs analyzed correspond to regions on nine different chromosomes (Table 1). The annotated genomic regions were used for a comparative analysis of gene order in relation to the lepidopteran reference genome (Figure 1), and for a comparative analysis of nucleotide sequence in relation to this and other lepidopteran species with relevant sequence available (Figure 2).

**Table 1:** Main characteristics of the *B. anynana* BACs analyzed.

BAC ID^1^	Target Gene^2^	Bany LG^3^	Length	GC content	% repeats	TE type^5^	KAIKO^6^	Validated^7^	# Unigenes^8^	Sequence Annotation^9^
(NCBI | CUGI | JGI)			(bp)	(%)	(S+LC)^4^				BAC/cds	(%)
AC239117 | 39A22 | 6132	ap	Z	98,442	36.1	0.3+1.9	-	35 (90)	1(182)	172/0	0.6/33.5/65.9
AC239122 | 69H15 | 6133	APC	6	127,416	35.7	0.4+1.9	-	32 (177)	4(678)	163/3	6.5/16.9/76.6
AC239115 | 19O01 | 6134	DP	4	126,420	34.7	1.2+2.6	NonLTR/RTE-3	36 (183)	10(449)	141/23	10.6/33.7/55.7
AC239118 | 39L19 | 6135	dpp	12	146,705	36.6	0.6+1.8	NonLTR/DMRT1	52 (123)	5(418)	132/9	4.3/19.9/75.8
AC239120 | 4N12 | 6136	EcR	10	97,772	36.3	0.4+2.0	DNA/Mariner	30 (122)	4(401)	180/10	4.9/65.9/29.2
AC239116 | 23M04 | 6137	en	2	109,203	36.7	0.6+2.7	LTR/BEL; Tc1-IS630-Pogo1	26 (166)	2(1582)	149/6	6.2/23.1/70.8
AC239119 | 48N20 | 6138	Dll	2	122,698	35.4	0.6+2.8	-	45 (82)	1(173)	153/3	0.4/11.7/87.8
AC239124 | 85J10 | 6139	nkd	5	121,582	35.3	0.3+2.2	-	28 (232)	6(595)	167/5	8.8/29.9/61.3
AC239114 | 18H03 | 6140	Sod2	3	108,600	36.1	0.6+3.0	NonLTR/RTE-3	42 (147)	11(275)	161/16	7.7/32.1/60.3
AC239121 | 68O14 | 6141	v	14	115,255	37.5	0.9+1.9	NonLTR/CR1; LTR/Gypsy	28 (225)	8(601)	120/22	12.5/42.3/45.2
AC239123 | 84B11 | 6142	Wg	4	129,178	36.7	0.5+2.2	NonLTR/RTE-1	44 (127)	3(480)	163/9	3.4/12.3/84.3

General characteristics observed for each BAC clone, including a summary of the annotation parameters discussed in the text.

1 BAC ID including NCBI accession number, BAC clone name from library at CUGI [27], and BAC sequence name from assembly by JGI. The CUGI and JGI names are used in the custom database [47];

2 Genes used to select the BACs for sequencing (see Introduction for abbreviations);

3 *B. anynana* linkage group based on mapping of the target genes (LG; [26]);

4 % repetitive sequence corresponding to single repeats (S) and low complexity (LC) regions as identified by RepeatMasker [41];

5 type of TEs, according to CENSOR [74] classification;

6 predicted peptides by Kaikogaas: number and (average aminoacid length);

7 predicted peptides after manual validation: number and (average aminoacid length);

8 Number of *B. anynana* UniGenes matching each BAC, and number corresponding to the validated predicted genes in Figure 1, cf. [47];

9 Percent sequence annotated as corresponding to protein-coding, intronic and intergenic DNA. Details in [47] and the supplementary files.

### CG content, repetitive sequence, and mobile elements

We used a combination of web-available and custom-designed bioinformatic tools and extensive manual curation to characterize different aspects of the target genomic sequence (details in Methods). Similar to observations in other lepidopterans [10], [37], [38], the GC content was ∼36.1% for the total sequence analyzed (with some variation between regions; Table 1) and ∼45.4% for the 55 validated predicted protein-coding genes (see below). This is consistent with studies in *Drosophila* where functional (coding) regions exhibit higher GC content than presumably less constrained regions [39], possibly relating to the fact that preferred codons often end in C or G [40].

We used RepeatMasker [41] to identify and characterize repetitive regions, including the type and extent of different repetitive elements. We identified a total of 857 repeats larger than 20 bp, corresponding to ∼2.73% (35567 bp) of the sequence analyzed (Table 1 and S1). The majority (721) of those repetitive elements were characterized as low complexity (i.e. poly-purine/poly-pyrimidine stretches or regions of >87% AT or >89% GC), and ∼0.49% of the total genomic sequence corresponded to simple repeats (duplications of, typically, 1–5 bp). While the overall ∼2.73% estimated repetitiveness for *B. anynana* is lower than the >20% estimate for *Heliconius* butterflies [10], [37], the proportion of that corresponding to low complexity repeats is higher than estimates for other lepidopteran species and the % of simple repeats is comparable between all [10], [37], [38]. Because our estimates are based on BACs selected for carrying specific genes, rather than sequence from randomly-selected BACs, it avoids gene-less regions and might under-estimate the extent of repetitiveness in the whole genome. Aside the repetitive elements identified by RepeatMasker, we also specifically looked for the nine novel types of repeated elements identified in other model butterflies [10] (see Methods). We found sequence similar to two of these in the available *B. anynana* genomic sequence (Table 1), and also in nucleotide sequences available for other lepidopteran species in NCBI's sequence depository (Table S2).

Using a combination of tools (RepeatMasker, Kaikogaas, CENSOR and manual BLAST; see Methods), we identified sequences related to transposable elements (TEs; Table 1). These included DNA transposons (Tc1-IS630-Pogo and DNA/Mariner), retroelements encoding for a reverse transcriptase (three NonLTR/RTEs, one NonLTR/DMRT and one NonLTR/CR1), and two LTR-retrotransposons (one LTR/BEL and one LTR/Gypsy) (see Table 1 and Figure 1). Only one of these nine TEs (Tc1-IS630-Pogo in BAC AC239116) was identified by RepeatMasker, and was, thus, within the sequence that was masked before further annotation (see Methods). This element is, thus, not in Figure 1 or Table S3. Some of the TEs identified are located inside introns (e.g., in *glycosyltransferase* in AC239115, and *Adh-4* in AC239114; Figure 1), and some are located near areas where synteny between *B. anynana* and *B. mori* is disrupted (e.g., inversion in AC239118, and transposition of *cytosolic ovarian carcinoma antigen 1* in AC239121; Figure 1). This is especially interesting because TEs are thought to play an important role in genome evolution, including contributing to chromosomal rearrangements [42] and to the appearance of new exons and introns (albeit possibly to a lesser degree in invertebrates [43]).

### *In silico* annotation and manual curation of protein-coding genes

For the *in silico* gene prediction we chose to use Kaikogaas [44], a web-available tool designed for annotation of genomic sequence of *Bombyx mori*, the lepidopteran reference [45]. This resulted in a total of 398 predicted peptides (Table 1 and S3). Of these, fewer than 10% (38) had any type of annotation (i.e. a putative gene name or function) beyond “hypothetical protein” (HP). To identify potential false positives and other issues with the *in silico* predictions, we manually curated the list of 398 Kaikogaas-derived genes extensively (details in Methods). In our conservative validation procedure we started by dismissing 111 predicted peptides shorter than 60 amino acids. We then used BLAST to check the remainder for sequence similarity in relation to relevant publicly-available gene collections. Only 55 predicted peptides (including the eight TEs not identified by RepeatMasker; see above) had significant similarity with proteins on NCBI and were kept for further analysis (Table 1, Figure 1 and Table S3). The curated 55 predicted genes in the 11 BAC sequences correspond to an average of 4.2 genes per 100 Kb which, despite our very conservative manual curation, falls well within published estimates for other insects (Figure S1), and is greater than that for other lepidopterans (∼2.5 genes/100 Kb in *Heliconius* butterflies [10] and ∼3.4 genes/100 Kb in the silkworm *B. mori* [46]). Note that gene density in the *B. mori* scaffolds orthologous to the available *B. anynana* sequence (the regions represented in Figure 1) is 4.5 genes/100 Kb, which is very close to that in *B. anynana*. Our manual curation strategy, designed to dismiss false positives at the expense of possibly generating false negatives, is expected to generate a conservative estimate of gene number. On the other hand, gene density in genomic regions selected for containing specific protein coding genes is probably higher than that in the whole genome as selection of gene-containing BACs avoids possible “gene deserts”.

The manual curation also allowed a more in-depth annotation of the predicted protein-coding genes. By annotating individual predicted exons we: 1) established that 41 of the 55 predicted genes had the complete putative coding sequences, 2) identified errors with the automated annotation process whereby exons of the same gene had been identified as different genes (e.g., Kaikogaas' HP12 gene in AC239120 corresponds to one of the exons of *Ecdysone receptor*; Table S3), and 3) highlighted instances of exon duplications, including cases of duplicated exons with (exons 11–15 of *p80 katanin* in AC239115, and *Brahma* in AC239121) and without (exons 2 and 3 of *Sod2* in AC239114, and exons 5 and 6 of *cell cycle progression* in AC239122) non-sense mutations (Table S3).

### Validation of *in silico* gene prediction using expressed sequence data

To match *in silico* gene predictions with expression data (see Methods), we used all *B. anynana* UniGenes (unique genes) from the available assembly of EST sequences [25], [26]. The results of this analysis are displayed graphically in a custom web-available database [47]. This approach allowed us not only to assess our annotation of protein-coding sequence, but also to assess EST assembly. We identified EST-derived UniGenes for most of our predicted peptides (Table S3), but also many UniGenes matching genomic regions with no predicted peptide (see [47]). We identified EST-derived UniGenes for 44 of the 55 predicted protein-coding genes; 17 with a single UniGene match and 27 with two to seven. In 23 of the 27 cases with more than one UniGene corresponding to the same predicted peptide, these UniGenes were at least partly overlapping. This reflects under-assembly of the ESTs, possibly due to polymorphisms in these sequences [25], [26], [48]. On the other hand, UniGenes in regions with no predicted peptides presumably correspond to false negatives of our conservative annotation. For example, Kaikogaas-predicted genes HP7 and HP35 in AC235115 and HP13 in AC239121 were discarded in the manual curation process (including one case under the 60-aminoacids long threshold). One UniGene corresponding to a putative transposable element not identified by Kaikogaas was detected in two BAC clones (AC239116 and AC239124). Still, the majority of UniGenes that did not match any predicted gene seemed to fall in repetitive regions identified and masked by RepeatMasker.

### *Alcohol dehydrogenase* expansion and sequence similar to lepidopteran miRNAs

**Figure 3:**
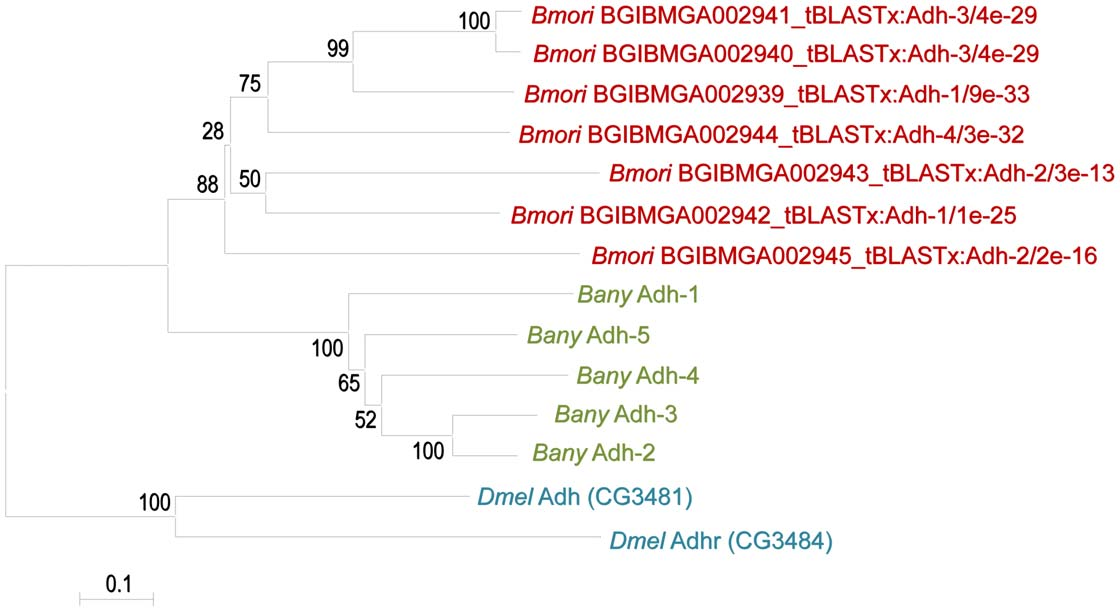
Phylogenetic tree of *Adh* genes. Neighbour-joining, unrooted tree reconstructed with MEGA 4 using the aminoacid sequence of the putative *Adh* genes in *B. anynana* (*Bany*, in green), together with the corresponding paralogs from chromosome 10 in *B. mori* (*Bmori*, in red, showing Silkdb gene accessions and BLAST results) and *D. melanogaster* (*Dmel*, in blue, showing FlyBase gene accessions). Numbers are bootstrap values for 1000 replicates.

We identified an interesting case of gene duplication. Among the predicted genes, we identified five putative *Alcohol dehydrogenase* (*Adh*) genes in tandem in BAC AC239114 (Figure 1). BLAST analysis (see Methods) allowed us to identify seven orthologs in chromosome 10 (BGIBMGA002939-45; minimal e-value 1e-30) of the *B. mori* lepidopteran reference genome, also annotated as *Adh* genes [49]. Phylogenetic analysis of the 12 corresponding Adh proteins, and including the Adh and Adh-related proteins of *D. melanogaster* as outgroups (see Methods), showed that all *B. anynana* paralogs cluster together and separated from the cluster of *B. mori* paralogs (Figure 3). This pattern of higher similarities within than between species may result from either of two types of scenarios: 1) independent duplications of *Adh* having occurred after the separation of the lineages of *B. mori* and *B. anynana*, or 2) duplications having occurred prior to the split of the lineages with subsequent concerted evolution of paralogs (gene conversion; see [50]). Unlike what has been shown for converted duplicates in other species [51], CG content in the five *B. anynana Adh* genes (average ± standard for entire loci is 36.1%±2.3%) and in the remaining 50 predicted genes (37.6%±6.2%) is not significantly different (t-test t = 0.521, df = 53, p = 0.60). This, however, does not distinguish between the scenarios above. More and other types of data are necessary for such purpose, and also for unravelling the ecological value of this gene expansion [52].

We used BLAST to search for *B. anynana* genomic sequence similar to mature miRNA sequences (∼22 nt) from *B. mori* available in miRBase [53], [54] and from *H. melpomene* [55] (see Methods). We identified a putative *B. anynana* miRNA similar to one of the 487 *B. mori* miRNAs (in BAC AC239117, Figure 1). Its sequence was 95% and 100% identical to that of its *B. mori* and *H. melpomene* counterparts, respectively. Prediction of miRNAs based on sequence conservation remains of limited value and needs experimental validation. The extent to which miRNAs are conserved is only now starting to be characterized. For example, only 68 of 257 *B. mori* miRNAs were found to be conserved with other species (23 with other vertebrates and invertebrates, 13 limited to invertebrates and 32 limited to insects [54]), and 430 of 447 chicken miRNAs were considered to be exclusive to the avian lineage [56]. In *Drosophila*, where species from the *Sophophora* and *Drosophila* subgenus diverged ∼62 MYA [57], only 28 of 59 *D. melanogaster* miRNA were conserved throughout the phylogeny of those species with fully sequenced genomes [58].

### Comparative analysis of gene order: fine-scale synteny with *B. mori*

The comparative analysis of gene order in the target *B. anynana* genomic regions (protein-coding gene annotation obtained as explained above) and the corresponding *B. mori* orthologous regions over 18 scaffolds (annotation available from SilkDB [49]) is represented in Figure 1. Note that both species have the same number of chromosomes and that *B. anynana* linkage groups were numbered following orthology with *B. mori* [26]. Some of the predicted 55 genes were excluded for a quantification of the synteny conservation: 1) three genes found isolated in three of the *B. anynana* BACs (AC239117, AC239116 and AC239119), 2) two genes whose *B. mori* orthologs were isolated in one scaffold (note that you need at least three gene pairs consecutive genes to assess conservation of order), and 3) the eight transposable elements in Figure 1. Out of 42 orthologous pairs analyzed, 36 genes (∼86%) are in the same order in both species. We also identified three *B. mori* genes (gray arrows in Figure 1) not represented in the corresponding *B. anynana* region (confirmed by running tBLASTn and tBLASTx of the *B. mori* sequence against the *B. anynana* BAC). One of these *B. mori* genes (BGIBMGA006231) presumably encodes a 57 amino acid protein with no SilkDB annotation [49] and might be a “false positive”. The other two (BGIBMGA010383 and BGIBMGA010570), however, are annotated and presumably encode longer peptides (776 and 304 amino acids, respectively). We used different BLAST algorithms to confirm that their absence in the corresponding *B. anynana* BAC was not the result of loss during our stringent manual curation. Interestingly, all three genes are associated with chromosomal rearrangements; one transposition in AC239115 and one inversion in AC239118 (Figure 1).

Previous results comparing genetic or physical maps had shown that chromosomal gene composition and gene order is highly conserved between butterflies and moths, but also revealed instances of chromosomal rearrangements [26], [59]. A fine-scale comparison of genomic sequence in ca. 420 Kb sequence between the butterfly *Heliconius erato* and the moth *B. mori* showed conserved gene order and distances for ca. 90% of the annotated protein-coding genes [10]. Here, in ca. 1303 Kb of sequence compared between *B. anynana* and *B. mori*, we detected smaller levels of conservation and identified 1) small scale inversions: inverted order of different genes (e.g., *mRpL46* and *dpp* in AC239118) and inverted direction of single genes (e.g., *p80 katanin* in AC239115), and 2) transpositions: with a number of genes assigned to non-orthologous chromosomes (e.g., *cytosolic ovarian carcinoma antigen 1* gene in AC239121 is in chromosome 11 in *B. anynana* and chromosome 14 in *B. mori*) and with the orthologs for the nine putative genes in AC239115 (*B. anynana* chromosome 1) distributed over five *B. mori* scaffolds, including regions in chromosome 4 and regions not yet assigned to a *B. mori* chromosome. Inversions and transpositions have been well documented between the sequenced genomes of *Drosophila* species [60], [61], [62] and are known to have played an important role in the evolution of their chromosomes.

### Comparative sequence analysis highlights conserved non-coding regions

Of the ca. 1,303 Kb of *B. anynana* genomic sequence analyzed, 6% corresponded to estimated coding sequence (cds), while 28% and 66% corresponded to predicted intronic and intergenic regions, respectively (Table 1). Using VISTA [63], [64], we compared the genomic sequence available for *B.anynana* with that of orthologous regions (identified as described in the Methods section) of *B. mori* and other Lepidoptera. The results are displayed in Figure 2. Note that for the putative *Adh* genes, even though the *B anynana* and *B. mori* copies showed conservation at the protein level (Figure 3), the VISTA software was unable to identify nucleotide sequence conservation in the corresponding regions. For all *B. anynana* regions compared, VISTA estimated a total of 4.3% nucleotide identity with *B. mori* (and 1.3% with butterflies *Papilio dardanus*, and 6.4% with *Heliconius melpomene*, for available sequence), with 2.8% in predicted coding region, 0.4% in introns, and 1.1% in intergenic regions (Table 1). Conserved sequence regions correspond largely to the location of putative exons (blue areas in Figure 2), but, in some instances, also to putative non-protein coding regions (red areas). Non-protein-coding DNA forms the majority of the genomes of many multicellular eukaryotes (e.g. ∼80% of noncoding DNA in *Drosophila* [65]), and ∼99% in humans [66]), and is known to be functionally important in many respects (e.g. for the regulation of gene expression and chromosome packaging). Conservation of non-coding sequence is often taken as a sign of possible functional importance. Different studies found considerable levels of conservation of non-coding sequence (e.g. between pairs of *Drosophila* species [67], [68], diverged no more than 62 MYA [57]) and constraints on intergenic regions have been estimated to possibly be as high as 60% [69].

The regions of significant conservation of non-coding sequence are heterogeneously distributed in the neighborhood of different genes (Figure 2). In fact, we see high levels of conservation around some (notably, *wingless* and *Ecdysone receptor*) targeted “developmental genes”, but not all (e.g. around *Distal-less* and *engrailed*). Like has been suggested for *Drosophila* where levels of selective constrain (and putative functional role) appear to correlate with intron length [70], *B. anynana wingless* and *Ecdysone receptor* have relatively large introns (∼13 Kb and ∼50 Kb, respectively), compared to the average of ∼6.5 Kb for the other 46 non-intronless predicted genes (their intron length ranging from 50 bp in the putative DNA/Mariner element to ∼33 Kb for the gene encoding transcription factor *apterous*). The high degree of conservation of non-coding sequence around *wingless* and *Ecdysone receptor* between *B. anynana* and other lepidopterans (∼78 MYA for *B. anynana* and *H. melpomene* [71]), and more than 140 MYA for *B. anynana* and *B. mori* [14]) is lost when comparing *B. anynana* to species in other insect orders (Figure S2). It is noteworthy that *wingless* and *Ecdysone receptor* are highly pleiotropic genes involved in a multitude of developmental processes across in multiple species, and which, in *B. anynana* are thought to be associated to the formation of characteristic wing color pattern elements [28] and seasonal polyphenism [72]. It will be interesting to extend this analysis to more lepidopteran species, including closer relatives with comparable wing pattern properties, and to explore the functional role of the conserved non-coding sequence experimentally.

### Overview and conclusions

We analyzed 11 BAC clones of the butterfly *Bicyclus anynana*, selected for the presence of key genes expressed during wing development at stages relevant for wing color pattern formation [25]. We have identified different genes in these regions, corresponding to gene densities similar to other lepidopterans. Among the genes identified, we discovered five tandemly arranged genes similar to *Alcohol dehydrogenase* (*Adh*), which potentially represent an expansion in the Lepidoptera. Comparative studies of sequence, expression and function of these genes are necessary to shed light onto their evolutionary history and ecological importance.

Our comparative analysis of the *B. anynana* genomic regions with the orthologous regions of the lepidopteran reference genome allowed assessment of conservation of fine-scale gene order and of DNA sequence. We detected strong synteny but 1) also multiple events where it was disrupted by different chromosomal rearrangements, including inversions and transpositions not detected with a previous comparative analysis of *B. anynana* versus *B. mori* linkage maps [26], and 2) lower proportion of genes in conserved order that a previous smaller-scale, fine-resolution analysis comparing *Heliconius erato* BAC-derived sequence with *B. mori* scaffolds [10]. We also detected instances of unusual high conservation of non-coding regions, in particular, around the genes encoding for Ecdysone Receptor and Wingless, between species diverged some 140 MYA. Understanding the functional significance of these regions will allow for a better understanding of the evolution and diversification of living organisms.

## Materials and Methods

### BAC sequences and target genes

We analyzed over 1,303,271 bp of nucleotide sequence from 11 *B. anynana* BAC clones deposited on GenBank (AC239114–AC239124). These BAC clones were obtained from the 9× coverage *B. anynana* BAC library available at Clemson University Genomics Institute (CUGI [27]) and were Sanger sequenced at 8–9× depth and their sequences assembled into 11 individual scaffolds by the Joint Genome Institute (JGI [73]), as part of the Community Sequencing Program FY2006. The sequenced BAC clones were originally selected by screening the BAC library filters with radioactively-labeled probes against 11 genes of interest (Table 1), expressed in developing wings [25].

### Annotation of genomic sequence

For the annotation of the BAC sequences aimed at characterizing different aspects of their genomic composition, we used a combination of web-available and custom-designed bioinformatic tools, as well as manual curation. We also used sequence information from orthologous regions in other species, with emphasis on the silkworm *B. mori*, the lepidopteran genomic reference.

RepeatMasker [41] with CrossMatch search algorithm (default settings) was used to identify repetitive regions, including the type and extent of different repetitive elements. The repetitive regions identified in this way were masked before all further analysis. Also, using BLASTn (e-value cut-off of 1e-5) against the *B. anynana* BAC sequences, we searched for nine transposable element families recently identified in another butterfly [10]. CENSOR [74] was used for classifying the identified putative transposable elements in the different classes (nonLTRs, LTRs, and DNA transposons) and, when possible, families.

*In silico* gene prediction was done for each complete BAC sequence using Kaikogaas (a web-available tool customized for *B. mori* genomic data; [44]). Kaikogaas' output is a graphical display (Figure S3) and a list of all putative proteins identified (Table S3) in each target BAC. This list was extensively manually curated for validation of Kaikogaas' predictions and for further annotation. Manual curation involved a sequence of steps: 1) hypothetical proteins shorter than 60 amino acids were dismissed; 2) predicted peptides longer than 60 amino acid were used to search for similarity with the complete collection of non-redundant insect protein sequences (this collection consisted of 913470 entries when our analysis was run in 2010) using BLASTp (e-value cut-off of 1e-10) and best hits were used for gene identification; 3) the amino acid sequences corresponding to these proteins were then used for running BLASTp (default settings) against the *Bombyx mori* genome in SilkDB [49], from where the corresponding scaffolds were downloaded (assembly of August 2010) and used for later comparison of genomic sequence around predicted genes (see below); 4) to attempt to confirm the putative exons of each hypothetical gene, we used homologs from both *B. mori* (from SilkDB [49]) and *D. melanogaster* (from Flybase [75]) to run tBLASTx (default settings) against the *B. anynana* BAC sequences.

BAC annotation was also done using a custom-built platform [47] which, aside repetitive regions and orthologs to genes in relevant public databases, also uses the complete list of UniGenes identified from the assembly of a large collection of *B. anynana* ESTs [25], [26]. BLAST results comparing the sequences of each complete BAC to the UniGene collection were deposited in a database queried by the GBrowse interface [76]. This analysis allowed, on the one hand, validation of the *in silico* prediction of exons, and, on the other, an assessment of EST assembly.

For the identification of conserved putative microRNAs (miRNAs), we ran BLASTn analysis (e-value cut-off of 1E-3; word size of 15) using as query against the *B. anynana* BAC sequences, the mature sequence (∼22 nt) of the 487 miRNA sequences of *B. mori* (available at miRBase [53], [54]) and the recently identified miRNAs from *Heliconius melpomene* butterflies [55].

### Comparative analysis of gene order and genomic sequence

To compare fine-scale physical linkage and gene order between *B. anynana* and *B. mori*, we used our annotation of the *B. anynana* BAC sequences (obtained as explained above) and the available annotation (SilkDB; [49]) for the corresponding *B. mori* scaffolds (identified as explained above, based on BLAST of predicted peptides during the manual curation).

To investigate nucleotide sequence conservation between *B. anynana* and other lepidopterans, we used VISTA (default settings: 70% identity, 100 bp window; [63], [64]) comparing whole *B. anynana* BACs with the corresponding genomic regions of other species. For *B. mori* these were obtained from the SilkDB scaffolds based on sequence similarity with the predicted *B. anynana* genes. For other species, they were obtained from BAC sequences available on NCBI, based on discontinuous megablast (default settings) of the complete *B. anynana* BAC sequence. Pairwise alignments between sequence for *B. anynana* sequence and each of the available corresponding sequences obtained were performed on the mVISTA program using the Avid alignment algorithm (default settings), which globally aligns DNA sequences of arbitrary length [77]. For the quantification of sequence conservation, we considered only the regions comprised between the first and last orthologous genes in each BAC that matched a single scaffold in the other species. The genomic regions around *EcR*, *wg* and *Wnt-6* (plus 15 Kb upstream and downstream) from *Drosophila melanogaster*, *Apis mellifera* and *Tribolium castaneum* were downloaded from Flybase [75], Beebase [78] and BeetleBase [79], [80], respectively. They were used for comparison with the same regions in *B. anynana* (AC239120 and AC239123) using VISTA (Figure S2).

### Phylogenetic analysis of *Adh* genes

Putative *Adh* genes from *B. anynana* (AC239114) were used for running tBLASTn and tBLASTx analysis against *B. mori* nucleotide and protein collection in SilkDB. The first hits corresponded to annotated *Adh* genes located at chromosome 10 of the silkworm. The corresponding proteins were downloaded, together with the ADH and ADHR (Adh-related) proteins of *D. melanogaster* from Flybase. These were used, together with the amino acid sequence of the putative *B. anynana* genes, for phylogenetic reconstruction using *MEGA* version 4 [81] (Neighbor-joining with pairwise deletion and bootstrap).

## Supporting Information

Table S1 **Quantification of repetitive sequence in target** ***B. anynana*** **BACs.** Identification and characterization of repeated regions was done with RepeatMasker (see Methods). Simple Repeats correspond to duplications of, typically 1–5bases. Low Complexity Sequence corresponds to poly-purine/poly-pyrimidine stretches or regions of >87% AT or >89% GC.
(PDF)

Table S2 ***Heliconius*** **repetitive elements identified in the target** ***B. anynana*** **BACs.** Two of the novel repetitive elements identified in *Heliconius* [10] appear to be present in our target *B. anynana* genomic sequence, as well as in publicly available nucleotide sequence for other lepidopterans (cf. BLASTn analysis; see Methods).
(PDF)

Table S3 **List of Kaikogaas predicted genes and subsequent manual curation.**
(TXT)

Figure S1 **Gene density and genome size in insects.** Gene density (number of genes per 100 Kb; *cf.* [10], [46], [82], [83], [84], [85], [86], [87], [88] in relation to genome size for different insect species (*cf.* [89]). Circles correspond to species where gene densities were estimated based on sequenced genomes – note that the size of assembled genome can differ from the estimates in this Figure. Other symbols correspond to species where gene density was estimated based on a few BAC clone sequences – including this paper for *B. anynana*.
(PDF)

Figure S2 **Comparative analysis of the** ***EcR*** **and** ***wg/Wnt-6*** **genomic regions.** VISTA plots of the genomic regions comprising the genes *EcR* (BAC AC239120) and *wg/Wnt-6* (BAC AC239123) with the orthologous regions in other insects with relevant sequence available: *Heliconius melpomene*, *Bombyx mori*, *Helicoverpa armigera*, *Spodoptera frugiperda*, *Drosophila melanogaster*, *Tribolium castaneum*, and *Apis mellifera*.
(PDF)

Figure S3 **Kaikogaas graphical output of BAC annotation.**
(PDF)
