# Supplementary material for: Genomic Sequence around Butterfly Wing Development Genes: Annotation and Comparative Analysis
Source: PLoS One. 2011 Aug 31;6(8):e23778. doi: 10.1371/journal.pone.0023778 (PMC3166123; doi:10.1371/journal.pone.0023778)
Supplement: Table S1 — Quantification of repetitive sequence in target B. anynana BACs. Identification and characterization of repeated regions was done with RepeatMasker (see Methods). Simple Repeats correspond to duplications of, typically 1–5bases. Low Complexity Sequence corresponds to poly-purine/poly-pyrimidine stretches or regions of >87% AT or >89% GC. (PDF) [file pone.0023778.s001.pdf]

Table S1. Quantification of repetitive sequence in target *B. anynana* BACs

**BAC ID** (GenBank)	**All repeats**			**Simple Repeats**			**Low Complexity Sequence**		
	**Length** (bp)	**%**	**Number**	**Length** (bp)	**%**	**Number**	**Length** (bp)	**%**	**Number** [rich in AT/GC/A/G/C/T]
AC239114	3189	2.94	76	655	0.60	14	2534	2.33	62 [59/0/0/2/1/0]
AC239115	3525	2.79	88	283	0.22	8	3242	2.56	80 [77/2/0/0/1/0]
AC239116	3928	3.60	78	627	0.57	15	2992	2.74	63 [57/0/4/1/1/0]
AC239117	2125	2.16	57	247	0.25	8	1878	1.91	49 [49/0/0/0/0/0]
AC239118	3499	2.39	85	864	0.59	16	2635	1.80	69 [65/1/0/0/1/2]
AC239119	4275	3.48	105	790	0.64	16	3485	2.84	89 [85/0/2/1/0/1]
AC239120	2324	2.38	53	411	0.42	8	1913	1.96	45 [42/1/1/0/1/0]
AC239121	3220	2.79	75	1012	0.88	19	2208	1.92	56 [52/2/0/1/1/0]
AC239122	2949	2.31	78	512	0.40	11	2437	1.91	67 [64/0/1/0/1/1]
AC239123	3445	2.67	81	640	0.50	10	2805	2.17	71 [69/1/0/0/1/0]
AC239124	3088	2.54	81	373	0.31	11	2715	2.23	70 [67/0/1/2/0/0]

Identification and characterization of repeated regions was done with RepeatMasker (see Methods). Simple Repeats correspond to duplications of, typically 1-5bases. Low Complexity Sequence corresponds to poly-purine/poly-pyrimidine stretches or regions of >87% AT or >89% GC.
