# Supplementary material for: Genomic Sequence around Butterfly Wing Development Genes: Annotation and Comparative Analysis
Source: PLoS One. 2011 Aug 31;6(8):e23778. doi: 10.1371/journal.pone.0023778 (PMC3166123; doi:10.1371/journal.pone.0023778)
Supplement: Table S2 — Heliconius repetitive elements identified in the target B. anynana BACs. Two of the novel repetitive elements identified in Heliconius [10] appear to be present in our target B. anynana genomic sequence, as well as in publicly available nucleotide sequence for other lepidopterans (cf. BLASTn analysis; see Methods). (PDF) [file pone.0023778.s002.pdf]

Table S2. *Heliconius* repetitive elements identified in the target *B. anynana* BACs

BAC	element 2				element 9			
	position	e-value	other leps	e-value	position	e-value	other leps	e-value
AC239122	29967-29997	2e-06	*Papilio dardanus*<br>*Helicoverpa armigera*<br>*Spodoptera littoralis*	1e-14<br>2e-13<br>6e-12	-	-	*Ostrinia nubilalis*<br>*Melitaea cinxia*<br>*Helicoverpa armigera*	5e-26<br>1e-09<br>3e-09
AC239115	56023-56068	2e-07			-	-		
	107905-107940	2e-07						
AC239118	76369-76405	1e-09			84559-84632	1e-06		
AC239120	18420-18453	2e-06			-	-		
	18263-18312	7e-06						
AC239119	65696-65768	2e-11			82849-82973	2e-06		
	30261-30297	5e-07			-	-		
AC239124	64714-64746	3e-10			-	-		
	64827-64874	4e-08						
AC239114	3091-3126	2e-06			-	-		
AC239121	38725-38797	5e-14			77007-77131	7e-06		

Two of the novel repetitive elements identified in *Heliconius* [10] appear to be present in our target B. anynana genomic sequence, as well as in publicly available nucleotide sequence for other lepidopterans (cf. BLASTn analysis; see Methods).
