# Supplementary material for: Genomic Sequence around Butterfly Wing Development Genes: Annotation and Comparative Analysis
Source: PLoS One. 2011 Aug 31;6(8):e23778. doi: 10.1371/journal.pone.0023778 (PMC3166123; doi:10.1371/journal.pone.0023778)

Figure S4. Gene density and genome size in insects. Gene density (number of genes per 100 Kb; *cf.* [10,46,82,83,84,85,86,87,88]) in relation to genome size for different insect species (*cf.* [89]). Circles correspond to species where gene densities were estimated based on sequenced genomes – note that the size of assembled genome can differ from the estimates in this Figure. Other symbols correspond to species where gene density was estimated based on a few BAC clone sequences – including this paper for *B. anynana* .

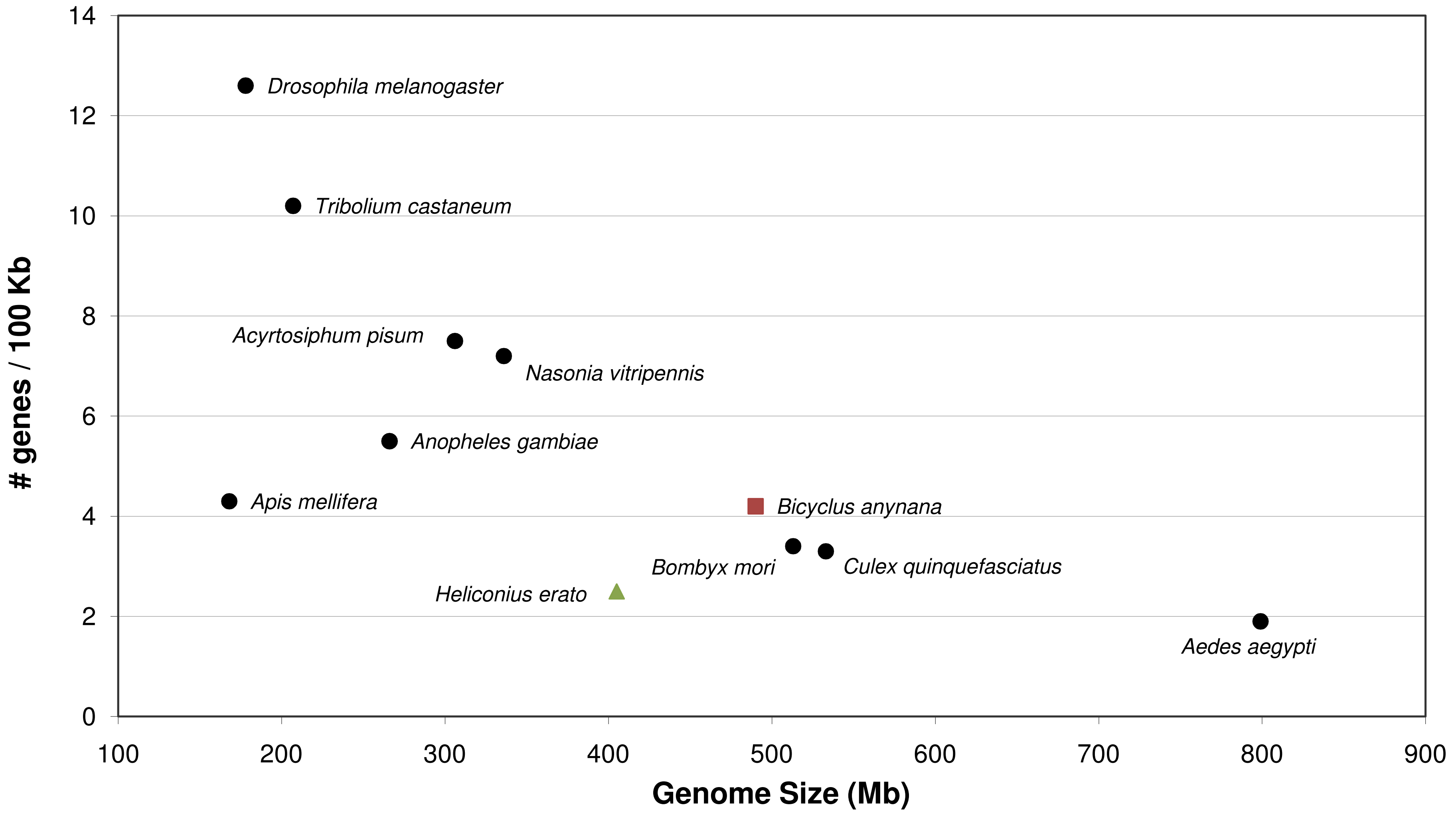

Supplement: Figure S1 — Gene density and genome size in insects. Gene density (number of genes per 100 Kb; cf. [10], [46], [82], [83], [84], [85], [86], [87], [88] in relation to genome size for different insect species (cf. [89]). Circles correspond to species where gene densities were estimated based on sequenced genomes – note that the size of assembled genome can differ from the estimates in this Figure. Other symbols correspond to species where gene density was estimated based on a few BAC clone sequences – including this paper for B. anynana. (PDF) [file pone.0023778.s004.pdf]
