# Supplementary material for: Genomic Sequence around Butterfly Wing Development Genes: Annotation and Comparative Analysis
Source: PLoS One. 2011 Aug 31;6(8):e23778. doi: 10.1371/journal.pone.0023778 (PMC3166123; doi:10.1371/journal.pone.0023778)

Figure S5. Comparative analysis of the *EcR* and *wg/Wnt-6* genomic regions. VISTA plots of the genomic regions comprising the genes *EcR* (BAC AC239120) and *wg/Wnt-6* (BAC AC239123) with the orthologous regions in other insects with relevant sequence available: *Bombyx mori*, *Drosophila melanogaster*, *Apis mellifera*, *Tribolium castaneum*, *Helicoverpa armigera*, *Spodoptera frugiperda,* and *Heliconius melpomene*.

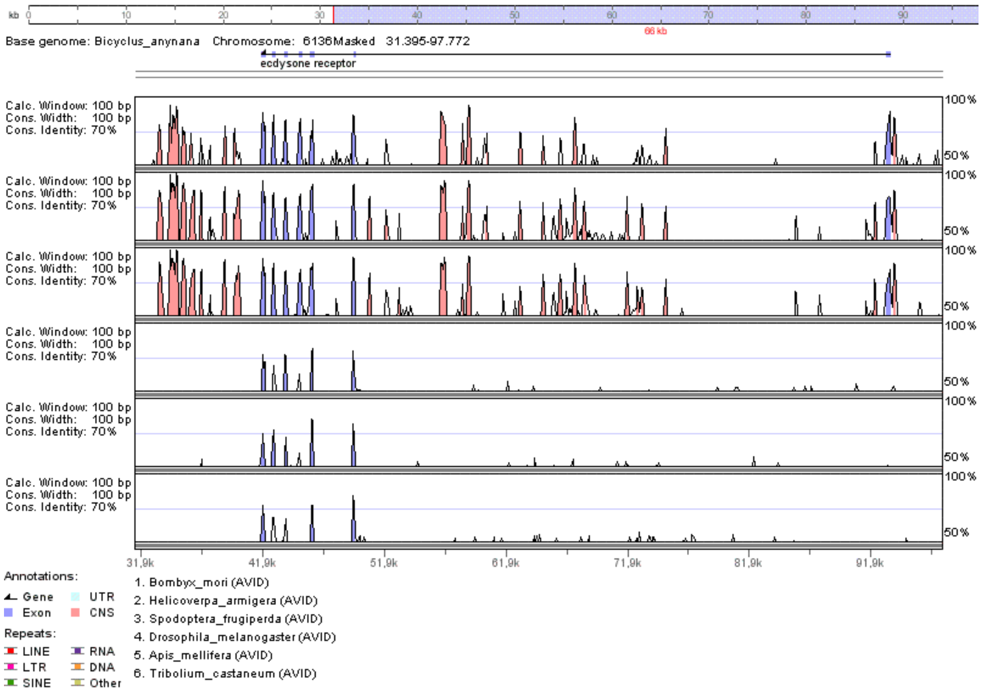

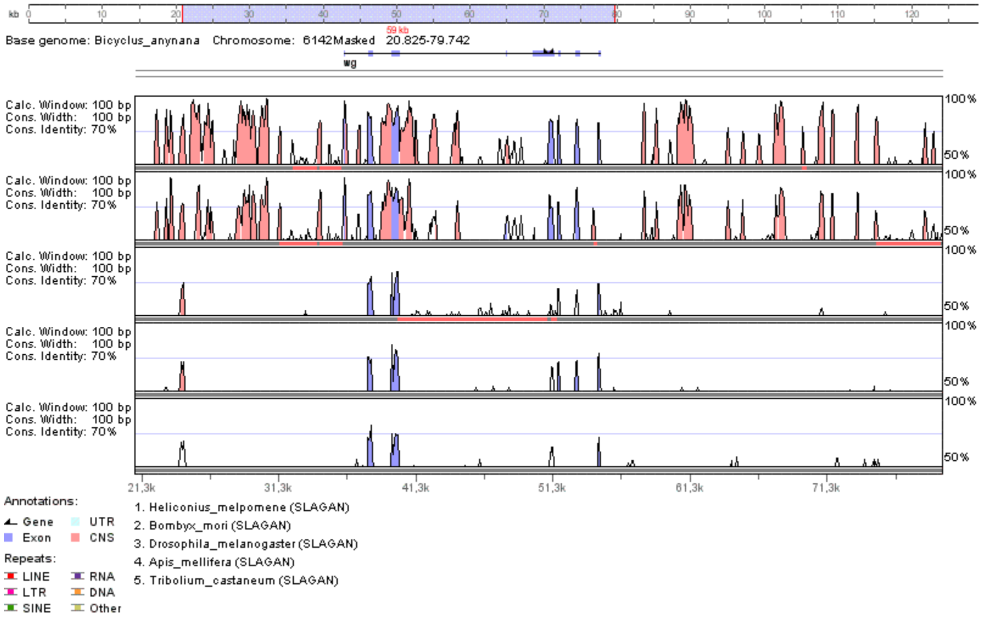

Supplement: Figure S2 — Comparative analysis of the EcR and wg/Wnt-6 genomic regions. VISTA plots of the genomic regions comprising the genes EcR (BAC AC239120) and wg/Wnt-6 (BAC AC239123) with the orthologous regions in other insects with relevant sequence available: Heliconius melpomene, Bombyx mori, Helicoverpa armigera, Spodoptera frugiperda, Drosophila melanogaster, Tribolium castaneum, and Apis mellifera. (PDF) [file pone.0023778.s005.pdf]
