# Supplementary material for: Genomic Sequence around Butterfly Wing Development Genes: Annotation and Comparative Analysis
Source: PLoS One. 2011 Aug 31;6(8):e23778. doi: 10.1371/journal.pone.0023778 (PMC3166123; doi:10.1371/journal.pone.0023778)
Supplement: Figure S3 — Kaikogaas graphical output of BAC annotation. (PDF) [file pone.0023778.s006.pdf]

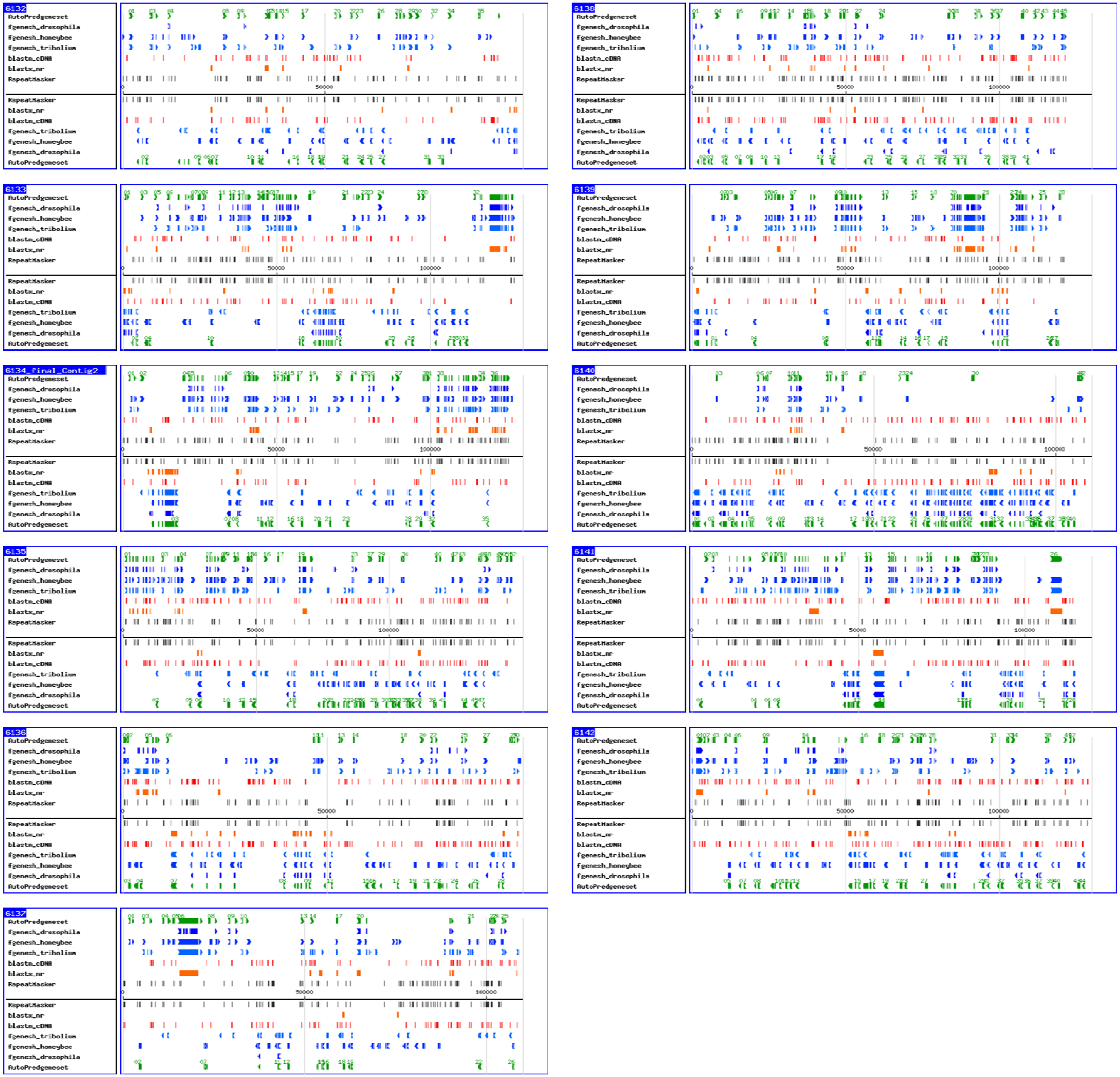

6132
6133
6134_final_Contig2
6135
6136
6137
6138
6139
6140
6141
6142
AutoPredgeneset
fgenesh_drosophila
fgenesh_honeybee
fgenesh_tribolium
blastn_cDNA
blastx_nr
RepeatMasker
50000
100000
